# Gene expression profiling following *NRF2* and *KEAP1* siRNA knockdown in human lung fibroblasts identifies CCL11/Eotaxin-1 as a novel NRF2 regulated gene

**DOI:** 10.1186/1465-9921-13-92

**Published:** 2012-10-12

**Authors:** Jimmy Fourtounis, I-Ming Wang, Marie-Claude Mathieu, David Claveau, Tenneille Loo, Aimee L Jackson, Mette A Peters, Alex G Therien, Yves Boie, Michael A Crackower

**Affiliations:** 1 Department of Respiratory and Immunology, Merck Research Laboratories, BMB10-128, 33 Avenue Louis Pasteur, Boston, Massachusetts, 02115, USA; 2 Exploratory and Translational Sciences, Merck Research Laboratories, West Point, Pennsylvania, USA; 3 Infectious Diseases, Merck Research Laboratories, Kenilworth, New Jersey, USA

**Keywords:** Asthma, NRF2, KEAP1, Oxidative stress, Eotaxin regulation, Microarray profiling

## Abstract

**Background:**

Oxidative Stress contributes to the pathogenesis of many diseases. The NRF2/KEAP1 axis is a key transcriptional regulator of the anti-oxidant response in cells. *Nrf2* knockout mice have implicated this pathway in regulating inflammatory airway diseases such as asthma and COPD. To better understand the role the NRF2 pathway has on respiratory disease we have taken a novel approach to define NRF2 dependent gene expression in a relevant lung system.

**Methods:**

Normal human lung fibroblasts were transfected with siRNA specific for NRF2 or KEAP1. Gene expression changes were measured at 30 and 48 hours using a custom Affymetrix Gene array. Changes in Eotaxin-1 gene expression and protein secretion were further measured under various inflammatory conditions with siRNAs and pharmacological tools.

**Results:**

An anti-correlated gene set (inversely regulated by *NRF2* and *KEAP1* RNAi) that reflects specific NRF2 regulated genes was identified. Gene annotations show that NRF2-mediated oxidative stress response is the most significantly regulated pathway, followed by heme metabolism, metabolism of xenobiotics by Cytochrome P450 and O-glycan biosynthesis. Unexpectedly the key eosinophil chemokine Eotaxin-1/CCL11 was found to be up-regulated when NRF2 was inhibited and down-regulated when KEAP1 was inhibited. This transcriptional regulation leads to modulation of Eotaxin-1 secretion from human lung fibroblasts under basal and inflammatory conditions, and is specific to Eotaxin-1 as *NRF2* or *KEAP1* knockdown had no effect on the secretion of a set of other chemokines and cytokines. Furthermore, the known NRF2 small molecule activators CDDO and Sulphoraphane can also dose dependently inhibit Eotaxin-1 release from human lung fibroblasts.

**Conclusions:**

These data uncover a previously unknown role for NRF2 in regulating Eotaxin-1 expression and further the mechanistic understanding of this pathway in modulating inflammatory lung disease.

## Background

Oxidative stress in tissues leads to the generation of reactive oxygen species which can interfere with normal cellular function and homeostasis and can contribute to the pathophysiology of many diseases including cancer, atherosclerosis, ischemia reperfusion injury, neurodegenerative disorders and aging [1]. The lung is highly susceptible to oxidant stress since it is exposed to high amounts of oxygen [2] and exogenous oxidants found in environmental pollution such as ozone or diesel exhaust particles [3]. As such, markers of oxidative stress are present in the lungs of people with many pathological conditions including asthma [4,5], COPD [6] and acute lung injury [7,8]. There is a large body of evidence from clinical and preclinical studies that this oxidative stress is a key contributor to the disease pathophysiology [9-17] and can modulate responses to pharmacological respiratory therapeutics [18].

Since oxidative stress can have such detrimental effects to the health of the organism, there has evolved an extensive endogenous intracellular and extracellular anti-oxidant system to maintain redox homeostasis [1]. One of the key regulators of this endogenous anti-oxidant system is the transcription factor nuclear factor (erythroid-derived 2)-like 2 (NFE2L2, NRF2). NRF2 is basic leucine zipper (bZIP) transcription factor that regulates the expression of numerous genes that encode anti-oxidant and detoxifying phase II enzymes through the binding to cis-acting anti-oxidant response elements (AREs) found in the promoters of these genes. Thus, NRF2 acts as the master regulator of the cellular response to oxidant injury [19]. In order to ensure that the anti-oxidant response is appropriately regulated, under conditions of redox homeostasis NRF2 is sequestered in the cytoplasm by binding through its N-terminal Neh2 domain to Kelch-like ECH-associated protein 1 (KEAP1) [20,21]. KEAP1 also functions as a substrate adaptor for the cullin-dependent E3 ligase and targets NRF2 for ubiquitination and degradation by the 26S proteasome [22,23]. Several stimuli including oxidants, toxic agents and electrophilic agents can lead to an oxidation of key sulphydryl groups on KEAP1 leading to the release of NRF2 where it can enter the nucleus and activate the anti-oxidant machinery [24,25]. In support of this, it has been shown that KEAP1 deficiency results in constitutive activation of NRF2 responsive gene expression [26].

There is significant data suggesting a critical role for NRF2 in preventing lung disease. Studies in COPD patients have shown that NRF2 dependent genes are activated in disease [27], but that as disease progresses there is a defect in this antioxidant response [28]. In preclinical species, there is increased expression of NRF2 regulated genes in cigarette smoke induced models of COPD [29] and in allergic lung models [17] implicating NRF2 as an endogenous regulator of oxidative stress in these models. This critical role has been confirmed in studies using *Nrf2* deficient mice. In an allergen-induced model of airway inflammation, loss of *Nrf2* has been shown to result in an increase in cellular recruitment to the lung, mucus hypersecretion and airway hyperresponsiveness [30]. Similarly, in cigarette smoke-induced models of COPD, *Nrf2* deficiency leads to an increase in inflammation and emphysema [31,32]. Additionally, *Nrf2* deficient mice have also been shown to have increased susceptibility to acute lung injury [33] and Respiratory Syncytial virus infection [34]. Importantly, treatment of mice with pharmacological agents that can activate NRF2 can lead to the inhibition of cigarette smoke [35] and allergen induced pathology in the lung [17]. Thus, there is a clear demonstration of the critical role of the endogenous anti-oxidant response and NRF2 in regulating airway disease.

In order to understand the precise mechanisms of the NRF2 induced anti-oxidant response, researchers have largely turned to expression profiling experiments to determine those genes that mediate NRF2 activity in the tissue or model of interest. Most of these studies have utilized *Nrf2* deficient mice or pharmacological treatment of various NRF2 activating compounds to define the NRF2 responsive genes [36-41]. These studies have lead to a well established group of NRF2 regulated genes, however, many novel or differentially regulated genes have been identified suggesting that there are species, tissue and model dependent differences in NRF2 regulated gene expression [42-47].

In this study we have taken a novel approach to define NRF2 dependent gene expression in normal primary human lung fibroblasts. These cells were chosen owing to the known role of oxidative stress pathways in fibroblasts [48], and the known role of fibroblasts to airway remodelling and a source of inflammatory mediators involved in asthma [49-51]. We have utilized siRNA to selectively and robustly knockdown the transcript levels of both *NRF2* and *KEAP1*. Using microarray profiling we have defined a distinct set of anti-regulated genes as well as genes specifically modulated by *KEAP1* or *NRF2* knockdown. Interestingly, we report the discovery that NRF2 activation by *KEAP1* knockdown or by pharmacological activators of NRF2 can specifically inhibit Eotaxin-1/CCL11 expression in human lung fibroblasts independent of several other chemokines further implicating this pathway in asthma pathogenesis.

## Methods

### Reagents

The IKK-β inhibitor Compound A was synthesized according to previously described methodology [52]. 2-cyano-3,12-dioxooleana-1,9(11)-dien-28-oic acid (CDDO) was synthesized according to previously described methodology [53]. Sulphorafane was purchased from Sigma Aldrich (Cat No. S6317). All siRNA pools were purchased from Sigma Proligo. The siRNA sequences are listed in Additional file 1. Two siRNA pools for *KEAP1* and all three pools for *NRF2* were comprised of 10 non-redundant siRNAs at low concentration (1 nM each) which has been shown to result in superior specificity while retaining potent target message knockdown compared to less complex pools at higher concentrations (our unpublished observations). The final pool for *KEAP1* was generated through the esiRNA technique [54].

**Table 1:** (A) Nrf2-mediated oxidative stress response genes knock down by *NRF2 *siRNAs and (B) Wnt/b-catenin signaling genes modulated by *KEAP1 *siRNAs

**GenBank**	**Name**	**Fold change**	**Description**
**A. NRF2-mediated Oxidative Stress Response**			
NM_001025433	NQO1	−5.24	NAD(P)H dehydrogenase, quinone 1
AY344083	TXNRD1	−4.1	thioredoxin reductase 1
NM_002133	HMOX1	−2.43	heme oxygenase (decycling) 1
NM_003900	SQSTM1	−1.73	sequestosome 1
NM_145792	MGST1	−1.71	microsomal glutathione S-transferase 1
L35546	GCLM	−1.63	glutamate-cysteine ligase, modifier subunit
NM_000637	GSR	−1.59	glutathione reductase
CR605580	FTH1	−1.56	ferritin, heavy polypeptide 1
NM_001757	CBR1	−1.52	carbonyl reductase 1
NM_000120	EPHX1	−1.3	epoxide hydrolase 1, microsomal (xenobiotic)
NM_003329	TXN	−1.25	thioredoxin
Y09188	FTL	−1.21	ferritin, light polypeptide
U30888	USP14	−1.17	ubiquitin specific peptidase 14 (tRNA-guanine transglycosylase)
NM_147148	GSTM4	−1.16	glutathione S-transferase M4
NM_181697	PRDX1	−1.16	peroxiredoxin 1
NM_000851	GSTM5	−1.15	glutathione S-transferase M5
NM_001315	MAPK14	−1.13	mitogen-activated protein kinase 14
M90656	GCLC	−1.08	glutamate-cysteine ligase, catalytic subunit
NM_000454	SOD1	−1.08	superoxide dismutase 1, soluble (amyotrophic lateral sclerosis 1 (adult))
NM_001752	CAT	−1.06	catalase
NM_003689	AKR7A2	−1.05	aldo-keto reductase family 7, member A2 (aflatoxin aldehyde reductase)
**B. Wnt/β-catenin Signaling**			
AF017987	SFRP1	−1.53	secreted frizzled-related protein 1
NM_014421	DKK2	−1.47	dickkopf homolog 2 (Xenopus laevis)
NM_012242	DKK1	−1.33	dickkopf homolog 1 (Xenopus laevis)
BC015915	FZD7	−1.29	frizzled homolog 7 (Drosophila)
L07590	PPP2R3A	−1.27	protein phosphatase 2 (formerly 2A), regulatory subunit B”, alpha
NM_003200	TCF3	−1.23	transcription factor 3 (E2A immunoglobulin enhancer binding factors E12/E47)
NM_000346	SOX9	−1.2	SRY (sex determining region Y)-box 9 (campomelic dysplasia, autosomal sex-reversal)
BC004372	CD44	−1.18	CD44 molecule (Indian blood group)
CR607734	DKK3	−1.18	dickkopf homolog 3 (Xenopus laevis)
NM_005634	SOX3	−1.18	SRY (sex determining region Y)-box 3
NM_005077	TLE1	−1.14	transducin-like enhancer of split 1 (E(sp1) homolog, Drosophila)
NM_001014794	ILK	−1.13	integrin-linked kinase
NM_000165	GJA1	−1.11	gap junction protein, alpha 1, 43 kDa
X70683	SOX4	−1.08	SRY (sex determining region Y)-box 4
BC114219	WNT3	2.09	wingless-type MMTV integration site family, member 3

### siRNA transfection and RNA preparation for microarray

Briefly, endoribonuclease-prepared short interfering RNAs (esiRNA) (*KEAP1*) or siRNA pools (10 individual siRNAs for each; pool 1: siRNA 1-10, pool 2: siRNA 11-20, pool 3: siRNA 21-30; see Additional file 1) for *NRF2* and *KEAP1* were incubated with Hiperfect reagent (Qiagen) in basal media (Lonza, # CC-3131) with no serum or antibiotics and allowed to complex for 10 min at room temperature. During this incubation, normal human lung fibroblasts (Lonza, #CC-2512) were plated in T25 flasks (6 × 10^5^ cells/flask) in media containing 2% serum and growth factors but no antibiotics. The complex was then added to the cell suspension of each well (final siRNA pool concentration of 10 nM, 1 nM of each siRNA). Cells were then incubated for 30 hr or 48 hr in a humidified incubator. At the end of the incubation period, the culture medium was removed and the cells were lysed by direct resuspension in Trizol reagent (Invitrogen, CA). Crude total RNA was isolated from Trizol-dissolved samples and purified using the RNAeasy kit (Qiagen) as per the manufacturer’s instructions. RNA concentration was measured using a NanoDrop ND-1000 (NanoDrop Technologies, Wilmington, DE), and RNA integrity was determined with a 2100 Bioanalyzer (Agilent Technologies, Santa Clara, CA). Samples displaying a RNA integrity number greater than 8 were used for profiling.

### Affymetrix GeneChip experiment

Samples were amplified and labelled using a custom automated version of the RT/IVT protocol and reagents provided by Affymetrix. Hybridization, labelling and scanning were completed following the manufacturer’s recommendations (Affymetrix). For data analysis, we used the mock-transfected sample as the reference to compare with all other time-matched samples to obtain the ratio data. Merck/Affymetrix human custom arrays monitoring 43,737 individual transcripts were used. Raw intensity was normalized using the RMA algorithm. Enrichment for biological processes was performed by comparing each gene signature against the public gene collections Gene Ontology, KEGG, Swissprot and Panther families. Enrichment P values (hypergeometric distribution) were corrected for multiple testing by using Bonferroni correction. Pathway analysis was performed using Ingenuity Pathway Analysis (IPA) (http://www.ingenuity.com/index.html). Overlap of each gene signature with other publicly available gene signatures was performed by using NextBio libraries (NextBio) (http://nextbio.merck.com/c/nextbio.nb).

### *NRF2* and *KEAP1* siRNA transfection for Q-PCR and chemokine/cytokine mesurements

Briefly, siRNA pools for *NRF2* and *KEAP1* were incubated with Hiperfect reagent (Qiagen, # 301705) in basal media (Lonza, # CC-3131) with no serum or antibiotics and allowed to complex for 10min at room temperature. During this incubation, normal human lung fibroblasts (Lonza, #CC-2512) were plated in 24-well or 96-well plates (Costar # 3524, BD Falcon # 353948), at 4×10^4^ or 2×10^4^ cells/well, respectively, with 2% serum but no antibiotics. The complex was then added to the cell suspension for each well (final siRNA pool concentration of 10 nM). Cells were then incubated for 48 hrs in a humidified incubator. After 48 hrs, cells were challenged with 1 ng/ml of human IL-1β (R+D systems # 201-LB-005) or PBS-0.1% BSA control for 18hrs. After 18 hrs challenge, cells were spun down for RNA isolation and supernatants were removed for cytokine and chemokine measurements.

### Real time quantitative PCR

Total RNA was isolated using QIAGEN RNeasy mini tubes according to the manufacturer’s animal cell extraction protocol (QIAGEN # 74106) which included the DNase step (QIAGEN # 79254). All TAQMAN probes were purchased from Applied Biosystems. Reverse transcription was performed in 100 μl of reaction solution using the following reagents per condition (Applied Biosystems # N808-234): 10 μl of 10X reverse transcription buffer, 20 μl of 25 mM MgCl_2,_ 10 μl of 10 mM dNTP mixture (2.5 mM each), 5 μl of 50 μM random hexamer, 5 μl of 20 U/μl RNase inhibitor, 5 μl of 50 U/μl Multiscribe reverse transcriptase and 45 μl of RNase-free H_2_O/RNA template mix. The RT-PCR reaction conditions 10min incubation at 25°C, 30min at 42°C and 5min at 99°C. The real time PCR reaction was carried out using the Fast TAQMAN PCR apparatus (Applied Biosystems) and the following reagents were used per PCR condition which was carried out in a 20 μl volume (all reagents purchased from Applied Biosystems except the water): 10 μl of 2X master mix, 1 μl of 20X TAQMAN primer-probe mix, 0.2 μl of AmpErase Uracil N-glycosylase, 0.8 μl of sterile water and 8 μl of cDNA template. The amplification conditions were as follows: 2 min at 50°C, 20 sec at 95C, followed by 40 cycles of 95°C for 1 sec and 60°C for 20 sec. All expression data was normalized for loading using human PPIA.

### Cytokine and chemokine measurements

Cells were cultured in the manner described above for siRNA knockdown studies. For studies using compounds, cells were seeded as described above, but in the absence of siRNA transfection. In this case, 1 day following plating, cells were treated with Compound A, Sulphorfane, CDDO or DMSO. 1 hour after compound dosing, cells were challenged with 1 ng/ml human IL-1β, or 10 ng/ml human TNFα R+D systems, # 210-TA), or 10 ng/ml mouse IL-13 or PBS-0.1% BSA control for an additional 24 hrs. Cells were then spun down and supernatants were assayed for cytokine and chemokine using Mesoscale Discovery platform assay plates (#K111AAB-2) according to manufacturer’s protocols.

### Statistical analysis

Student’s *t* test was performed on all data points. All data are represented as mean ± Standard Deviation.

## Results

### siRNA knockdown of *NRF2* and *KEAP1* in NHLFs

**Figure 1:**
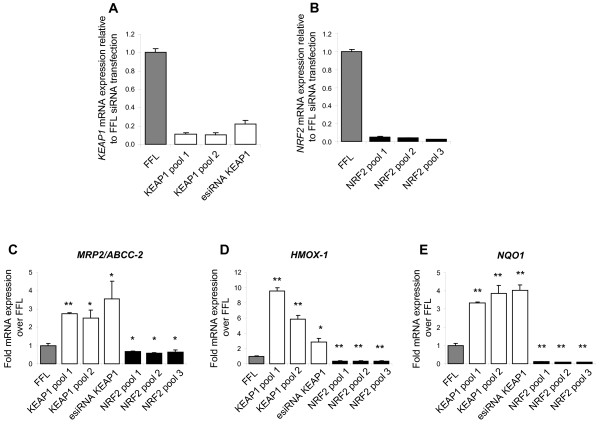
**Changes in mRNA expression following *NRF2 *and *KEAP1 *gene-specific siRNA transfection in NHLFs.** (**A,C-E**) Cells were transfected with negative control siRNA targeting firefly luciferase (FFL), and 2 pools of 10 siRNAs (*KEAP1* pool 1 and pools 2) or an esiRNA preparation targeting *KEAP1* (esiRNA *KEAP1*) and evaluated for *KEAP1*, *MRP2/ABCC-2*, *HMOX1* and *NQO1* mRNA expression by QPCR. (**B-E**) Cells were transfected with negative control siRNA targeting firefly luciferase (FFL), and 3 pools of 10 siRNAs targeting *NRF2* (*NRF2* pool 1, pool 2, pool 3) and evaluated for *NRF2, MRP2/ABCC-2*, *HMOX1* and *NQO1* mRNA expression by QPCR. Data is expressed as expression levels relative to the FFL transfection. n=4 for each data point. A student’s t-test was performed on each data set relative to the FFL data (*p < 0.5, **p < 0.01). Data are represented as mean ± standard deviation.

To better understand NRF2/KEAP1 regulated genes in the lung, we chose to employ siRNA knockdown in normal human lung fibroblasts (NHLFs) to specifically modulate this pathway. In this approach, we utilized knockdown of *KEAP1*, which should result in NRF2 activation [26], to identify those genes regulated by NRF2 activation and utilized knockdown of *NRF2* to better define those genes dependent on baseline NRF2 activity. To minimize any confounding effects of potential off-target activity of siRNA [55] we conducted our study using three distinct pools of siRNA for both *KEAP1* and *NRF*2. As shown in Figure 1, significant knockdown (> 80%) of both *KEAP1* (Figure 1A) and *NRF2* (Figure 1B) mRNA was achieved for all pools tested, as measured by quantitative PCR (QPCR), compared to the negative control firefly luciferase siRNA transfection. To ensure that knockdown of *NRF2* and *KEAP1* in NHLFs resulted in a significant modulation of classical NRF2 regulated genes we analysed the transcript levels of the ARE-regulated genes *MRP2*, *HMOX-1* and *NQO1* following transfection at both time points by (QPCR). *KEAP1* knockdown resulted in a significant upregulation of the expression of all of the genes tested at both time points indicating that NRF2 is activated as a result of *KEAP1* knockdown (Figure 1C-E). Interestingly, *NRF2* knockdown resulted in a decrease in the basal expression of all of these genes (Figure 1C-E) showing that basal activity of NRF2 is required for the expression of these genes in non-stressed conditions. Overall these data indicate that this siRNA approach resulted in significant functional modulation of the KEAP1/NRF2 pathway.

### Gene expression profiling following *NRF2* and *KEAP1* siRNA knock-down

**Figure 2:**
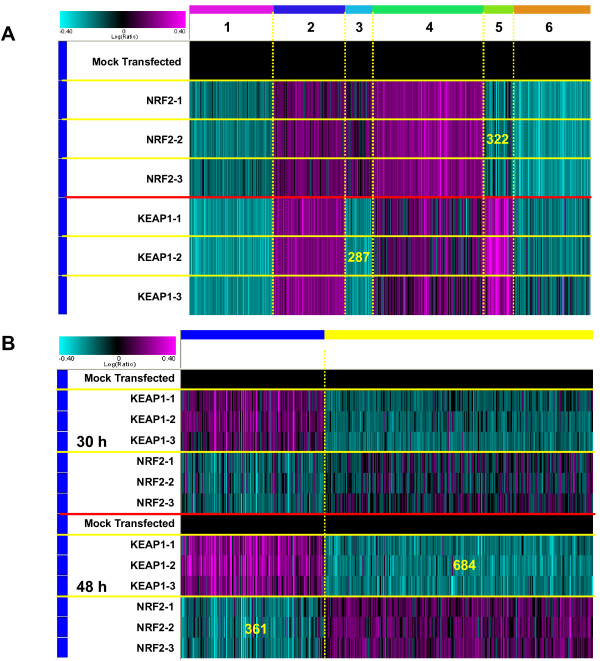
***K-means *clustering of gene signature modulated by either *KEAP1 *or *NRF2 *siRNAs.** (**A**) *K-means* clustering of gene signature modulated by either *KEAP1* or *NRF2* siRNAs at 48 hours post-transfection. Three replicates were profiled for each siRNA pool. One way ANOVA (*NRF2* or *KEAP1* siRNA-transfected vs. mock-transfected samples) was performed to identify genes up- or down-regulated by *NRF2* or *KEAP1* siRNA at *p* ≤ 0.01. Data from all three replicates of each siRNA pool were combined *in silico* and further filtered by absolute fold change ≥ 1.15. (**B**) *K-means* clustering of the anti-correlated gene signature modulated by *KEAP1* and *NRF2* siRNAs. The gene signature was obtained by combining anti-correlated genes modulated by *KEAP1* and *NRF2* siRNAs at 30 hours (308 genes at replicates combined p<=0.05) and 48 hours (893 genes at replicates combined p<=0.05). We further removed a 43 interferon-inducible gene set resulting from a KEAP1-3 siRNA pool to obtain a final signature gene set of 1,045 sequences with 361 sequences down-regulated by *NRF2* siRNAs and 684 sequences down-regulated by *KEAP1* siRNAs.

To define genes regulated by the NRF2/KEAP1 pathway in human lung fibroblasts we conducted microarray mRNA profiling 30 and 48 hours following *NRF2* and *KEAP1* siRNA knockdown. For each siRNA pool, 3 replicates were profiled. ANOVA analyses were then performed to identify genes up- or down-regulated by *NRF2* or *KEAP1* siRNA at p value of less or equal to 0.01. Data from all three replicates of each siRNA pool were combined and a further filter by absolute fold change of more than or equal to 1.15 was applied. With these filtering criteria, the expression of 2,729 and 2,136 sequences, accounting for 6.2% and 4.9% of the transcriptome probed on our arrays, was significantly modulated by *NRF2* and *KEAP1* knockdown, respectively. *NRF2* siRNA knockdown resulted in the down-regulation of **1,139** sequences and the up-regulation of **1590** sequences. *KEAP1* knockdown resulted in the down-regulation of **1175** sequences and the up-regulation of **961** sequences. Figure 2A shows a k-means clustering of the union signature of either *NRF2* or *KEAP1* siRNA modulated genes. Most of the *NRF2* or *KEAP1* siRNA modulated genes are up- (clusters 2 and 4, with 754 and 1,184 sequences, respectively) or down-regulated (clusters 1 and 6, with 892 and 834 sequences, respectively) in a consistent manner. Annotation of the up-regulated genes (clusters 2 and 4; total 1,938 sequences) by both *NRF2* and *KEAP1* siRNA indicated an association with multiple developmental processes, including cardiovascular, skeletal, neural and muscular systems; also affected are the cytoskeletal organization, extracellular matrix, apoptosis and WNT signaling pathways (Additional file 2). *NRF2* and *KEAP1* siRNA down-regulated genes (clusters 1 and 6; total 1,726 sequences) are mainly associated with cell cycle progression/regulation, DNA replication and repair (Additional file 3).

With this analysis approach, two gene clusters (e.g. clusters 3 and 5 with 287 and 322 sequences, respectively) are differentially regulated by *KEAP1* and *NRF2* siRNAs.

### Selecting and annotating anti-correlated *NRF2* and *KEAP1* siRNA knock-down genes

To identify those genes whose expression was inversely regulated when comparing *NRF2* and *KEAP1* knockdown, genes were selected if they were modulated in the opposite direction by *NRF2* and *KEAP1* siRNA with combined p values less than 0.05 at 30 and 48 hours after siRNA transfection. We observed 113 common sequences between 308 and 893 signature sequences obtained from 30 and 48 hour time points; in addition, we removed a 43 gene signature specifically induced by the pool of 3 *KEAP1* siRNA which is highly enriched in interferon-responsive genes and is most likely a property of that particular pool of siRNAs. Figure 2B shows a K-means clustering of the resulting 1,045 sequences which met the selection criteria with 361 sequences and 684 sequences down-regulated by *NRF2* and *KEAP1* siRNA, respectively. Lists of most highly down- and up-regulated genes by NRF2 siRNA at 48 hours can be found in Additional file 4.

We then queried the biological processes and pathways associated with the 893 sequences (from 48 hour time point) using resources from GO Biological Process (see Additional file 5) and Ingenuity Pathways. Additional file 6 shows Ingenuity canonical pathway analysis of the gene set derived from anti-correlated genes knocked down by *NRF2* and *KEAP1* siRNA, respectively. Genes involved with the most significant pathways affected by the 2 siRNA treatments (i.e. NRF2-mediated oxidative stress response and Wntβ-catenin signalling) are listed in Table 1. It is interesting to note that several Wnt/β-catenin signalling pathway genes were down-regulated by *KEAP1* siRNA with the exception of *WNT3* which was up-regulated 2.1 fold.

### Eotaxin-1 expression is suppressed with *KEAP1* siRNA knockdown

**Figure 3:**
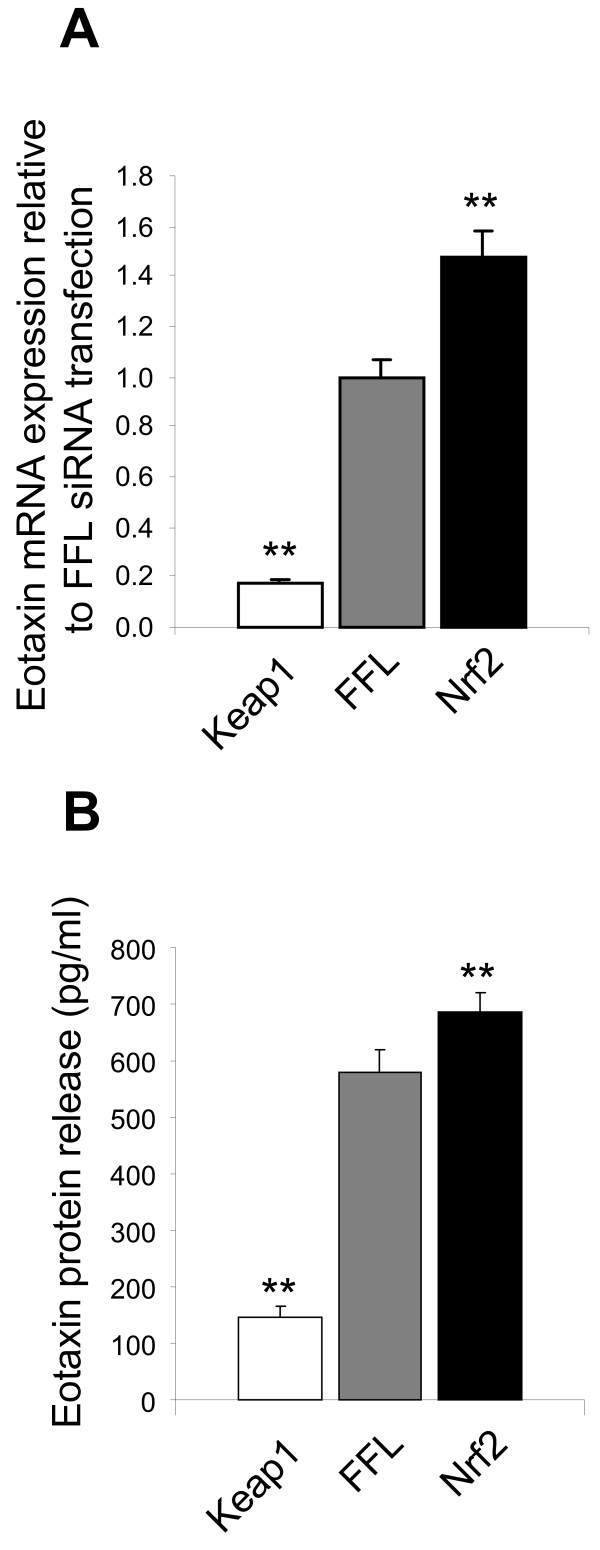
**Modulation of Eotaxin-1 expression following *KEAP1 *and *NRF2 *siRNA knockdown.** Cells were transfected with negative control siRNA targeting firefly luciferase (FFL), 1 siRNA pool targeting *KEAP1* (KEAP1), and 1 siRNA pool targeting *NRF2* (NRF2) and evaluated for (**A**) mRNA expression and (**B**) secreted potein levels of Eotaxin-1. Data is expressed levels relative to the FFL transfection. n=4 for each data point. A student’s t-test was performed on each data set relative to the FFL data (*p<0.5, **p<0.01). Data are represented as mean ± standard deviation.

In the microarray profiling, we observed that CCL11/Eotaxin-1 a key chemokine for eosinophil recruitment to the lung [56], is regulated by the KEAP1/NRF2 pathway. Knockdown of *KEAP1* led to a suppression of Eotaxin-1 expression, whereas knockdown of *NRF2* lead to an increase in Eotaxin-1 levels. Regulation of Eotaxin-1 has not been previously reported in gene expression profiling studies of the NRF2/KEAP1 axis. Therefore to confirm this observation we independently transfected NHLFs with *KEAP1* or *NRF2* siRNA and indeed confirmed by QPCR (Figure 3A) that upon knockdown of *KEAP1* baseline Eotaxin-1 mRNA level was reduced approximately 80% relative to control siRNA transfection. Conversely, upon knockdown of *NRF2* baseline Eotaxin-1 mRNA level was increased approximately 50% relative to control siRNA transfection. To determine if these changes resulted in modulation of Eotaxin-1 protein levels secreted from the NHLFs we evaluated levels of Eotaxin-1 protein in the media from these siRNA knockdown experiments. Similar to the changes in Eotaxin-1 mRNA expression, we did find that knockdown of *KEAP1* results in a significant decrease of secreted Eotaxin-1 levels from NHLFs, whereas a significant increase in Eotaxin-1 release was observed with *NRF2* siRNA transfection (Figure 3B).

### *KEAP1* knockdown specifically inhibits Eotaxin-1 in NHLFs under inflammatory conditions

**Figure 4:**
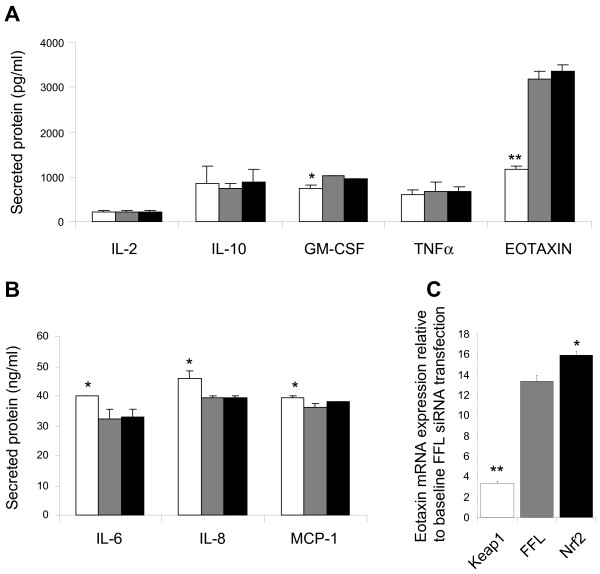
**Modulation of IL-1β induced cytokine/chemokine secretion following *KEAP1 *and *NRF2 *siRNA knockdown.** Cells were transfected with negative control siRNA targeting firefly luciferase (FFL), 1 siRNA pool targeting *KEAP1* , and 1 siRNA pool targeting *NRF2*. (**A,B**) 24 hours following transfection cells were stimulated with IL-1β and select cytokine and chemokine release was evaluated*.* Data is expressed as protein levels found in tissue culture supernatants. n=2 for each data point. (**C**) Eotaxin-1 mRNA expression was also evaluated following IL-1β challenge. Data is expressed as mRNA levels relative to baseline FFL transfection ( see Figure 5). N=4 for each data point. A student’s t-test was performed on each data set relative to the FFL data (*p<0.5, **p<0.01). Data are represented as mean ± standard deviation.

In addition to the role of the KEAP1/NRF2 pathway in regulating the anti-oxidant response, it has also been shown that activation of NRF2 can have profound anti-inflammatory effects [57]. We thus sought to evaluate the regulation of Eotaxin-1 by KEAP1/NRF2 under inflammatory conditions. To this end, we challenged NHLFs with IL-1β to induce an inflammatory response and evaluated the secretion of several cytokines and chemokines including Eotaxin-1. Treatment with IL-1β resulted in a significant increase in, Eotaxin-1, IL-2, IL-10. GM-CSF, TNFα, IL-6, IL-8, and MCP-1. Interestingly when NHLFs were transfected with *KEAP1* siRNA prior to IL-1β challenge very modest increases in IL-6, IL-8 and MCP-1 secretion (Figure 4B) were observed, and a very modest decrease in GM-CSF was observed (Figure 4A). On the other hand a significant reduction of secreted Eotaxin-1 levels were observed (appox. 63%) upon *KEAP1* knockdown (Figure 4A). Unlike the effects of *NRF2* knockdown observed at baseline, no significant increase of Eotaxin-1 release was observed by *NRF2* knockdown upon IL-1β challenge. However, when mRNA expression changes were analysed, a counter regulation of Eotaxin-1 mRNA expression was observed with IL-1β challenge similar to effects at baseline (Figure 4C).

**Figure 5:**
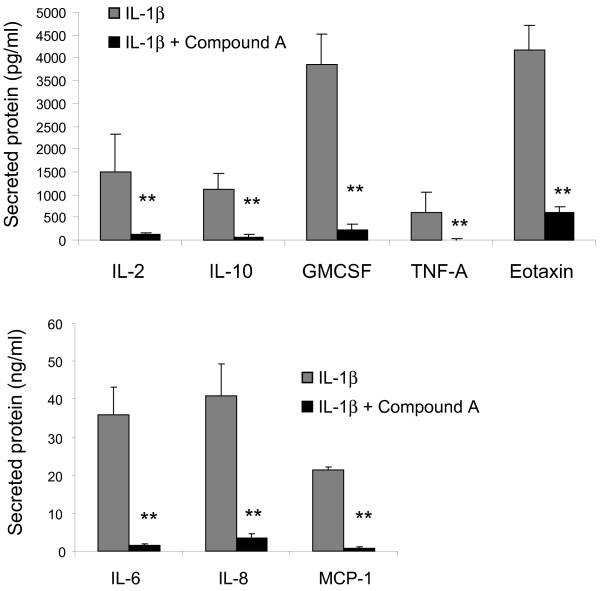
**Modulation of IL-1β induced cytokine/chemokine by the IKK-β inhibitor Compound A **[61]**.** Cells were treated with Compound A for 24 hours prior to IL-1β stimulation. Following IL-1β challenge select cytokine and chemokine release was evaluated. Data is expressed as protein levels found in tissue culture supernatants. n=3 for each data point. A student’s t-test was performed on each data set relative to the untreated data set (*p<0.5, **p<0.01). Data are represented as mean ± standard deviation.

NRF2 activation is thought to lead to the inhibition of NF-κB activity [58]. NF-κB is a broad pro-inflammatory mechanism that can regulate the activity of multiple secreted cytokines and chemokines including Eotaxin-1 [59,60]. Thus it is possible that the suppression of Eotaxin-1 observed with *KEAP1* knockdown is simply mediated by the inhibition of NF-κB activity. To investigate this, we treated NHLFs with a potent and selective IKK-β inhibitor (Compound A) [61] prior to stimulation with IL-1β. Treatment with 1 μM of compound A had profound and robust effects on the secretion of all of the cytokines induced by IL-1β including Eotaxin-1 (Figure 5). The selective inhibition of Eotaxin-1 by *KEAP1* knockdown argues that the mechanism by which NRF2 activation is modulating Eotaxin-1 expression is not simply through the inhibition of NF-κB activity.

### NRF2 activating compounds sulforaphane and CDDO specifically suppress IL-1β, IL-13 and TNFÎ± induced Eotaxin-1 in NHLFs

**Figure 6:**
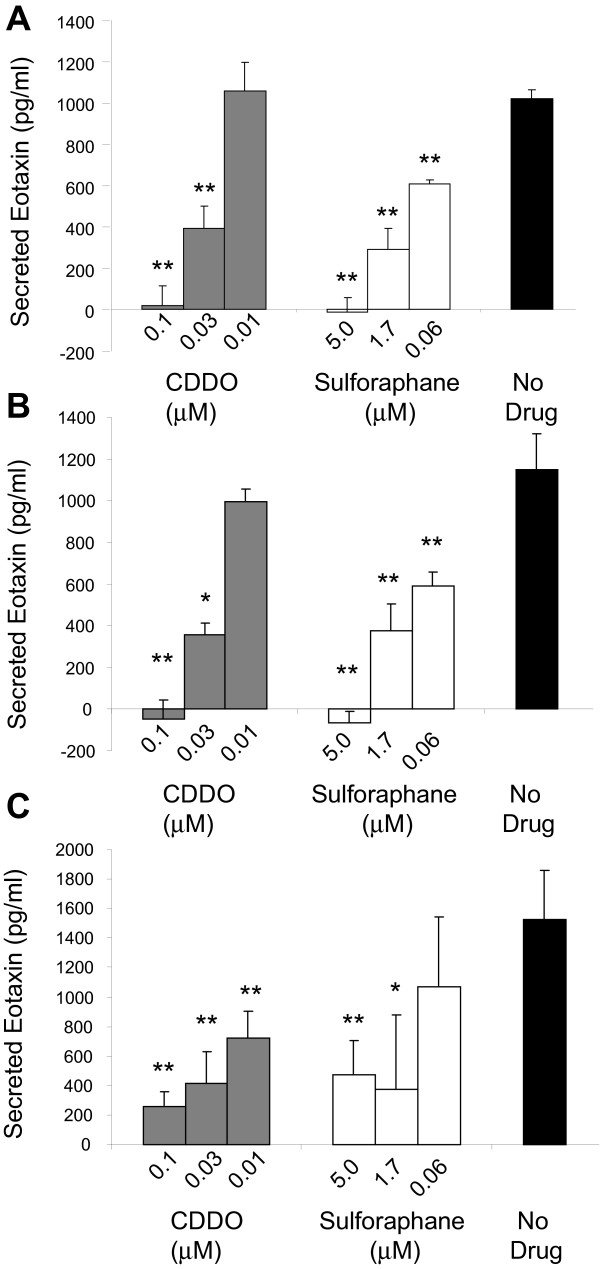
**Modulation of IL-1β, TNFα and IL-13 induced Eotaxin-1 release by CDDO and Sulforaphane.** Cells were treated with Compound 1 hour prior to stimulation. Secreted Eotaxin-1 was measured 24 hours following challenge. (**A**) Dose dependent inhibition of IL-1β induced Eotaxin-1 release. (**B**) Dose dependent inhibition of TNFα induced Eotaxin-1 release. (**C**) Dose dependent inhibition of IL-13 induced Eotaxin-1 release. Data is expressed as protein levels found in tissue culture supernatants. n=3 for each data point. A student’s t-test was performed on each data set relative to the untreated data set (*p<0.5, **p<0.01). Data are represented as mean ± standard deviation.

Several pharmacologic agents have been shown to activate NRF2. These include the dietary isothiocyantes sulforaphane [47] and the synthetic triterpenoid CDDO [62]. Since siRNA can have off-target effects we used these pharmacological modulators of NRF2 activity to evaluate their effect on Eotaxin-1 expression in NHLFs. Similar to siRNA knockdown of *KEAP1*, treatment with sulforaphane or CDDO resulted in a significant dose-dependent decrease in Eotaxin-1 secretion following IL-1β challenge (Figure 6A). This data provides further confirmation that indeed Eotaxin-1 is specifically inhibited by NRF2 activation in NHLFs. To further explore the role of NRF2 in Eotaxin-1 release under inflammatory conditions, we challenged NHLFs with IL-13 and TNFα following treatment with CDDO and sulforaphane. Similar to IL-1β, IL-13 and TNFα lead to a robust induction of Eotaxin-1 release from fibroblasts (Figure 6B and C). Treatment with CDDO and sulforaphane also led to a dose dependent decrease in Eotaxin-1 release under these conditions (Figure 6B and C). These data suggest that NRF2 activation can inhibit Eotaxin-1 release from lung fibroblasts under diverse inflammatory conditions.

## Discussion

Here we present our results of microarray profiling of normal human lung fibroblast following siRNA mediated knockdown of *NRF2* and *KEAP1*. We have identified a distinct gene set of anti-correlated genes in this analysis to better define NRF2 regulated genes in a lung specific cellular context.

A comparison of the 1,045 signature sequences differentially modulated by *NRF2* and *KEAP1* siRNA (Figure 2) with other gene expression signatures collected in the Gene Expression Omnibus (GEO. http://www.ncbi.nlm.nih.gov/geo/) data base indicates a highly significant anti-correlation with a gene signature obtained from primary human lung fibroblast treated with dithiothreitol (DTT) for 24 hours (Overlapping P = 1.7E-99) [63]; and a significant correlation with a gene set from dexamethasone-treated (24 hours) human primary osteoblast-like cells (HOb)(GEO series GSE10311.Overlapping P = 1.0E-46). In addition, we found two cigarette smoke-related gene signatures which are anti-correlated to our gene signature, one from a normal human bronchial epithelial (NHBE) cells exposed to a cigarette smoke condensate for 18 hours (Overlapping P = 2.5E-33, data derived from GEO series GSE18235 [64], and the other from a comparison of small airway epithelial cells between smokers and non-smokers (overlapping p = 2.3E-24) [65]. Since DTT and cigarette smoke induce ER stress and oxidative stress, respectively; it appears that NRF2 is activated in both situations to confer cellular protection.

In addition to NRF2 promoting the anti-oxidant response machinery, this pathway also has profound anti-inflammatory effects [57]. Studies with NRF2 deficient mice demonstrate an increased inflammatory response in several inflammatory disease models [58,66,67]. In respiratory models, the loss of *Nrf2* results in increase eosinophil recruitment in the lungs of allergen challenged animals and the increase in lung macrophages upon hyperoxic lung injury [33]. In models of COPD, *Nrf2* deficient mice have increased neutrophil and macrophage recruitment to the lung [31]. In vitro studies have demonstrated a specific effect of the NRF2 regulating cytokine and chemokine expression in neutrophils following LPS challenge [68]. In addition, pharmacological activation of NRF2 with the triterpenoid CDDO can inhibit LPS induced inflammation in neutrophils and PBMCs [68].

In this study we make the novel discovery that Eotaxin-1 is uniquely inhibited by NRF2 activation. While the direct role of NRF2 on Eotaxin-1 regulation has not be reported previously, mice deficient for *Nrf2* do have increased eosinphil recruitment to the lung upon allergen challenge associated with increased level of Eotaxin-1 in the BAL fluid [30]. In addition, it has been demonstrated that mice with a deficiency of NADPH oxidase in non-hematopoietic cells have decreased lung eosinophilia during allergen challenge implicating the ROS in the production of Eotaxin-1 in the lung [9]. Interestingly, it has been shown that dietary flavonoids inhibit Eotaxin-1 release from fibroblasts [69,70]. Flavonoids have various anti-inflammatory properties and are potent inhibitors of NF-κB signalling [71] but are also potent activators of NRF2 [72]. This inhibition of Eotaxin-1 observed is consistent with our study where we show inhibition of Eotaxin-1 with the triterpenoid CDDO. Based on our data with *KEAP1* knockdown it can be concluded that the inhibitory effect that these flavonoids have on Eotaxin-1 is likely mediated directly by their activation of NRF2 and not through other anti-inflammatory mechanisms.

As the major eosinophil chemoattractant, Eotaxin-1 plays a critical role in allergic inflammation and asthma [73]. In the lung Eotaxin-1 promotes the influx of eosinophils where activation and release of key mediators of an inflammatory response occurs [56]. The role of the fibroblast in mediating eosinophil recruitment has long been established [74]; where it has been shown that fibroblasts derived from numerous sources secrete a significant amount of Eotaxin-1 in response to several pro-inflammatory stimuli [75-79]. Consistent with this, we have demonstrated in this report that IL-1β, IL-13 and TNFα all have potent effects on Eotaxin-1 secretion in fibroblasts. These factors are key inducers of Eotaxin-1 release and eosinophil recruitment in addition to contributing to fibrotic changes seen in airway disease [75,79]. It would be of interest to evaluate an NRF2/ Eotaxin-1 relationship in fibroblasts from asthmatics to determine if Eotaxin-1 expression would be equally regulated by NRF2 activation is a disease state.

The mechanism by which Eotaxin-1 is modulated by NRF2 is not known. A detailed promoter study failed to identify a bonafide ARE [80] upstream of the human Eotaxin-1 gene, suggesting that this inhibition may be an indirect consequence of NRF2 activation. One way in which NRF2 has been shown to mediate its anti-inflammatory properties is through the inhibition of NF-κB. NRF2 and NF-κB have been shown to work together to modulate inflammatory gene expression [58,60] and it has been suggested that NRF2 activation can lead to NF-κB inhibition [58,81-83]. In addition it has been shown that the NF-κB pathway plays a critical role in Eotaxin-1 regulation in fibroblasts [30,59]. While it is not clear if this is the case in our study, it is unlikely since we have demonstrated using pharmacological inhibition that all of the chemokines and cytokines induced by IL-1β and TNFα are NF-κB dependent, yet only Eotaxin-1 is inhibited by NRF2 activation.

Another key transcription factor that can mediate Eotaxin-1 expression is STAT6. A STAT6 binding site is present on the Eotaxin-1 promoter along with an NF-κB binding site and it is thought that Eotaxin-1 may be regulated by the concerted activity of NF-κB and STAT6 [84]. STAT6 is of course a key mediator of Eotaxin-1 expression induced by IL-4 [85], but studies in fibroblasts have shown that STAT6 also is required for TNFα induced Eotaxin-1 expression [86]. Thus, it remains feasible that in someway, NRF2 activation inhibits STAT6 activity, thus leading to the inhibition of Eotaxin-1 expression. There is no published data directly linking NRF2 activation to STAT6 activity, however, in one study using the licorice root triterpenoid Glycyrrhizin, it has been demonstrated that inhibition of Eotaxin-1 with this compound is associated with the inhibition of STAT6 phosphorylation and nuclear translocation [87]. This data suggests that perhaps NRF2 does indeed regulate Eotaxin-1 expression through the regulation of STAT6 activity. Another potential mechanisms by which NRF2 may modulate Eotaxin-I expression is through modulation of MAPK signaling as it has been demonstrated that MAPK signaling downstream of TGFβ can synergize with IL-13 to induce Eotaxin-1 expression by interfering with negative feedback loops in the IL-13/STAT6 pathway [51]. Interestingly it has been demonstrated that reactive oxygen species can directly augment the activity of STAT6 [88] raising the possibility that a decrease in reactive oxygen species as a result of NRF2 activation may inhibit STAT6 activity and inhibit Eotaxin-1 expression.

## Conclusions

In summary, through gene expression profiling of normal human lung fibroblasts, following siRNA knockdown of *NRF2* and *KEAP1*, we have identified Eotaxin-1 as a novel NRF2 regulated gene. Our data further define the role of this pathway in mediating inflammatory disease in the lungs.

## Abbreviations

NRF2: Nuclear factor (erythroid-derived 2)-like 2; KEAP1: Kelch-like ECH-associated protein 1; CDDO: 2-cyano-3,12-dioxooleana-1,9(11)-dien-28-oic acid; COPD: Chronic obstructive pulmonary disease; ARE: Anti-oxidant response elements; siRNA: Small interfering Ribonucleic acid; IKK-β: Inhibitor of kappa light polypeptide gene enhancer in B-cells, kinase beta; esiRNA: Endoribonuclease-prepared short interfering RNAs; RNA: Ribonucleic acid; RT/IVT: Reverse transcription/in vitro transcription, reverse transcription/in vitro; RNAi: RNA interference; Q-PCR: Quantitative polymerase chain reaction; NHLFs: Normal human klung fibroblasts; NHBE: Normal human bronchial epithelial cells; OVA: Ovalbumin; IL: Interleukin.

## Competing interests

I-M W, DC, AGT and MAC are full time employees of Merck and Co.

## Authors’ contributions

MAC, AGT and YB were responsible for experimental design. MAC, JF and I-MW were responsible for data analysis and manuscript preparation. MCM performed all siRNA KD studies. DC conducted TAQman studies. JF and TL conducted experiments assessing Eotaxin expression. ALJ and MAP designed and synthesized esiRNA pools. All authors read and approved the final manuscript.

## Supplementary Material

Additional file 1 **Sequences of siRNAs.** List of siRNA sequences utilized for gene expression knockdown studies. Click here for file

Additional file 2 **Upregulated genes by NRF2 and KEAP1 siRNA knockdown.** List of genes whose expression is increased with NRF2 and KEAP1 siRNA knockdown. Genes are group based on annotated biological processes. Click here for file

Additional file 3 **Down-regulated genes by NRF2 and KEAP1 siRNA knockdown.** List of genes whose expression is decreased with NRF2 and KEAP1 siRNA knockdown. Genes are group based on annotated biological processes. Click here for file

Additional file 4 **Most significant *NRF2*- and *KEAP1*-modulated anti-correlated genes identified in microarray studies.** Figure displaying (A) Top 30 genes knockdown by *NRF2* siRNAs at 48 hours. Genes were sorted based on their fold change knocked down by *NRF2* siRNAs at 48 hours. The corresponding fold changes modulated by *NRF2* siRNAs at 30 hours and *KEAP1* siRNAs at 48 and 30 hours are also shown. (B) Genes significantly knock down by *KEAP1* siRNAs at 48 hours. Genes were sorted based on their fold change knocked down by *KEAP1* siRNAs at 48 hours. The corresponding fold changes modulated by *NRF2* siRNAs at 30 and 48 hours and *KEAP1* siRNAs at 30 hours are also shown. CCL11/eotaxin is included in the Figure as a reference although it is not one of the top 30 *KEAP1* siRNAs knock down genes. Click here for file

Additional file 5 **Annotation of anti-correlated NRF2 and KEAP1 siRNA knock-down gene set at 48 hours.** List of genes whose expression is modulated in an anti-correlated direction with NRF2 and KEAP1 siRNA knockdown. Genes are group based on annotated biological processes. Click here for file

Additional file 6 **Ingenuity pathway analysis of anti-correlated genes knock-down by (A) *NRF2 *siRNAs or (B) *KEAP1 *siRNAs.** Ingenuity pathway analysis of anti-correlated genes knock-down by (A) *NRF2* siRNAs or (B) *KEAP1* siRNAs. Of the 1,045 anti-correlated signature genes, 361 sequences down-regulated by *NRF2* siRNAs and 684 sequences down-regulated by *KEAP1* siRNAs were individually up-loaded onto Ingenuity Pathway Analysis (IPA) tool for querying canonical pathways associated with the input gene sets (http://www.ingenuity.com). Click here for file
