# Supplementary material for: Gene expression profiling following NRF2 and KEAP1 siRNA knockdown in human lung fibroblasts identifies CCL11/Eotaxin-1 as a novel NRF2 regulated gene
Source: Respir Res. 2012 Oct 12;13(1):92. doi: 10.1186/1465-9921-13-92 (PMC3546844; doi:10.1186/1465-9921-13-92)
Supplement: Additional file 1 — Sequences of siRNAs. List of siRNA sequences utilized for gene expression knockdown studies. [file 1465-9921-13-92-S1.pdf]

Additional file 1. Sequences of siRNAs. Sequence are displayed 5'-3'. m indicates 2' -O-methyl modification at this residue.

POOL #	Oligo Name	Sense Sequence	Antisense sequence
NRF2-1	NFE2L2-1	mGmAUUCUGACUCCGGCAUUUTT	AmAAUGCCGGAGUCAGAAUCTT
	NFE2L2-2	mCmACUACUUGGCCUCAGUGATT	UmCACUGAGGCCAAGUAGUGTT
	NFE2L2-3	mCmUCACAAGAGAUGAACUUATT	UmAAGUUCAUCUCUUGUGAGTT
	NFE2L2-4	mGmAGAUGAACUUAGGGCAAATT	UmUUGCCCUAAGUUCAUCUCTT
	NFE2L2-5	mGmCUCAUACUUUAUAAGUAATT	UmUACUUAUAAAGUAUGAGCTT
	NFE2L2-6	mGmAGACUACCAUGGUUCCAATT	UmUGGAACCAUGGUAGUCUCTT
	NFE2L2-7	mCmUGUUGAUUUAGACGGUAUTT	AmUACCGUCUAAAUCAACAGTT
	NFE2L2-8	mGmCCCUCACCUGCUACUUUATT	UmAAAGUAGCAGGUGAGGGCTT
	NFE2L2-9	mCmCAUUCACUCUCUGAACUUTT	AmAGUUCAGAGAGUGAAUGGTT
	NFE2L2-10	mGmUCACUUGUUCCUGAUAUUTT	AmAUAUCAGGAACAAGUGACTT
NRF2-2	NFE2L2-11	mCmUGAAAGCACAGCAGAAUUTT	AmAUUCUGCUGUGCUUUCAGTT
	NFE2L2-12	mGmAAAGCACAGCAGAAUUCATT	UmGAAUUCUGCUGUGCUUUCTT
	NFE2L2-13	mCmAGAGAAAGAAUUGCCUGUTT	AmCAGGCAAUUCUUUCUCUGTT
	NFE2L2-14	mGmUAAGUCCUGGUCAUCGGATT	UmCCGAUGACCAGGACUUACTT
	NFE2L2-15	mCmAGAAGUUGACAAUUAUCATT	UmGAUAAUUGUCAACUUCUGTT
	NFE2L2-16	mCmUACUUGGCCUCAGUGAUUTT	AmAUCACUGAGGCCAAGUAGTT
	NFE2L2-17	mGmAUGUGAAAUGCUCAUACUTT	AmGUAUGAGCAUUUCACAUCTT
	NFE2L2-18	mCmUAUCACUUUGCAAAGCUUTT	AmAGCUUUGCAAAGUGAUAGTT
	NFE2L2-19	mCmAGUGAUUCUGAAGUGGAATT	UmUCCACUUCAGAAUCACUGTT
	NFE2L2-20	mCmAAGUUUGGGAGGAGCUAUTT	AmUAGCUCCUCCCAAACUUGTT
NRF2-3	NFE2L2-21	mGmUGAGAACACACCAGAGAATT	UmUCUCUGGUGUGUUCUCACTT
	NFE2L2-22	mCmAGUCUUCAUUGCUACUAATT	UmUAGUAGCAAUGAAGACUGTT
	NFE2L2-23	mCmUGAAACUUCUGUUGCUCATT	UmGAGCAACAGAAGUUUCAGTT
	NFE2L2-24	mCmUGUUGUUGACUUCAACGATT	UmCGUUGAAGUCAACAACAGTT
	NFE2L2-25	mGmAGUAAGUCGAGAAGUAUUTT	AmAUACUUCUCGACUUACUCTT
	NFE2L2-26	mCmAUGCUACGUGAUGAAGAUTT	AmUCUUCAUCACGUAGCAUGTT
	NFE2L2-27	mGmACAUUCCCGUUUGUAGAUTT	AmUCUACAAACGGGAAUGUCTT
	NFE2L2-28	mCmUUGCAUUAAUUCGGGAUATT	UmAUCCCGAAUUAAUGCAAGTT
	NFE2L2-29	mCmUCAGUUACAACUAGAUGATT	UmCAUCUAGUUGUAACUGAGTT
	NFE2L2-30	mCmAAUGAAGCUCAACUUGCATT	UmGCAAGUUGAGCUUCAUUGTT
Keap1-1	KEAP1-1	mCmGAAUGACAUCGGGCCGGATT	UmCCGGCCCGAUGUCAUUCGTT
	KEAP1-2	mGmUGUUACGACCCAGAUACATT	UmGUAUCUGGGUCGUAACACTT
	KEAP1-3	mCmCUUAAUUCAGCUGAGUGUTT	AmCACUCAGCUGAAUUAAGGTT
	KEAP1-4	mCmAGAUUGGCUGUGUGGAGUTT	AmCUCCACACAGCCAAUCUGTT
	KEAP1-5	mGmCUAUGAUGGUCACACGUUTT	AmACGUGUGACCAUCAUAGCTT
	KEAP1-6	mGmGUUCUACGUCCAGGCGCUTT	AmGCGCCUGGACGUAGAACCTT
	KEAP1-7	mGmCAUCAACUGGGUCAAGUATT	UmACUUGACCCAGUUGAUGCTT
	KEAP1-8	mGmGGACAAACCGCCUUAAUUTT	AmAUUAAGGCGGUUUGUCCCTT
	KEAP1-9	mCmGCCUUAAUUCAGCUGAGUTT	AmCUCAGCUGAAUUAAGGCGTT
	KEAP1-10	mCmGAAUGAUCACAGCAAUGATT	UmCAUUGCUGUGAUCAUUCGTT
Keap1-2	KEAP1-11	mCmAACUUCGCUGAGCAGAUUTT	AmAUCUGCUCAGCGAAGUUGTT
	KEAP1-12	mGmAGUACAUCUACAUGCAUUTT	AmAUGCAUGUAGAUGUACUCTT
	KEAP1-13	mGmAAACAGAGACGUGGACUUTT	AmAGUCCACGUCUCUGUUUCTT
	KEAP1-14	mGmACAAACCGCCUUAAUUCATT	UmGAAUUAAGGCGGUUUGUCTT
	KEAP1-15	mCmUCAUUGAAUUCGCCUACATT	UmGUAGGCGAAUUCAAUGAGTT
	KEAP1-16	mGmCAAUGAACACCAUCCGAATT	UmUCGGAUGGUGUUCAUUGCTT
	KEAP1-17	mCmGAACUUCCUGCAGAUGCATT	UmGCAUCUGCAGGAAGUUCGTT
	KEAP1-18	mCmAGAUUGACCAGCAGAACUTT	AmGUUCUGCUGGUCAAUCUGTT
	KEAP1-19	mGmUCAUGUACCAGAUCGACATT	UmGUCGAUCUGGUACAUGACTT
	KEAP1-20	mGmUAUGAGCCAGAGCGGGAUTT	AmUCCCGCUCUGGCUCAUACTT
