# Supplementary material for: Gene expression profiling following NRF2 and KEAP1 siRNA knockdown in human lung fibroblasts identifies CCL11/Eotaxin-1 as a novel NRF2 regulated gene
Source: Respir Res. 2012 Oct 12;13(1):92. doi: 10.1186/1465-9921-13-92 (PMC3546844; doi:10.1186/1465-9921-13-92)
Supplement: Additional file 2 — Upregulated genes by NRF2 and KEAP1 siRNA knockdown. List of genes whose expression is increased with NRF2 and KEAP1 siRNA knockdown. Genes are group based on annotated biological processes. [file 1465-9921-13-92-S2.pdf]

Similar Set	Expectation	Overlap	Set	Input Identifiers
cardiovascular system development	6.31E-25	124	718	PPAP2B;FBN1;PLCG1;ITGB8;ZFPM2;CD40;PKD1;LAMA4;ANGPTL2;VEGFB;ATP7A;TCF7L2;HOXA3;COL5A1;LRP6;PTCH1;CD248;TCF25;ERBB4;CDH2;FN1;ELN;ADAMTS15;HDAC5;ADAM19;COL18A1;NAA15;SLIT2;MED13L;WARS2;FOXP4;GPI;DICER1;SIN3B;SOX9;MMP14;TIPARP;TGFB2;CTNNB1;GLI3;NTN4;ZMIZ1;WARS;SEMA5A;PLCD3;EDNRA;SP1;STAT3;ACVRL1;CREB1;MAP2K5;PTN;HTR2B;FZD4;WASF2;PRKD2;LRP5;ITGA5;IL22RA1;RHOB;NF1;EFNB2;COL1A1;DCBLD1;EP300;COL4A1;ECM1;LMNA;COL6A1;ZFAND5;HRG;ECE1;SMAD3;GPR124;CDC42;SDC3;RAD50;RECK;ERBB2;PLXND1;SRC;MYH9;SOX4;FZD2;TBK1;PKD2;ERAP1;TPM1;ROBO1;POU6F1;COL3A1;TSC1;ADRA1B;COL15A1;THBS2;TGFBR3;VCAN;CYR61;COL8A2;LBH;BMP4;TBX5;CASP7;EPAS1;CPE;PTPLA;EPHB4;RB1CC1;QKI;CTGF;MMP19;TGFBR2;THBS1;MYLK;GATA6;WNT5A;PLXDC2;GYS1;MIB1;HDAC7;KRIT1;FIGF;TNC;DLC1
cytoskeleton organization	8.67E-14	117	881	JMY;FSCN1;CLIP3;LIMK1;FGD6;PRKG1;BRWD3;CDC14B;NAV1;ARAP3;PLEK;ARHGEF17;FERMT2;WASF1;MACF1;SYNPO;COL18A1;MAP2;SLIT2;DST;GCC2;ARHGEF3;PLK2;RICTOR;ABLIM3;EPB41L1;EHD2;CLIP1;AKAP9;AGRN;ROCK2;CTNNB1;PTPRF;MARCKS;NTN4;WARS;CMIP;MAP3K1;KANK1;STAT3;SEPT11;SGCD;BCL6;FHOD1;ARHGAP17;WASF2;KIRREL;TACC1;SORBS3;PRKCI;PALLD;SHROOM2;ARPC2;RHOB;NF1;TRPM7;EP300;MAP1B;ANTXR1;CDC42BPB;HRG;LLGL1;RABGAP1L;SMAD3;PTPRK;NF2;CALD1;KIF3B;CDC42;TNIK;VAPB;INF2;LATS2;SDC3;ARHGAP20;RAD50;MYL9;SRC;MYH9;CDC25B;ROCK1;SSH1;SMC3;AKAP13;EVL;TPM1;DISC1;EHBP1;SVIL;MID1;TSC1;AFAP1;ABI2;LIMA1;THBS2;DNAH5;TTLL3;SPTAN1;CXCL12;FGD4;MYO9B;ARAP1;PLD1;RANBP2;EPHB4;DAAM1;CTGF;TRIOBP;NEFH;ST5;MYH10;FLNB;DNM3;TLN2;TNC;DLC1;SUN1
skeletal system development	2.35E-11	62	356	RPS6KA3;PDGFRB;FBN1;PCGF2;HOXB8;PKD1;SATB2;LUM;ATP7A;HOXA3;SIX1;DICER1;SOX9;EXT1;TIPARP;CTNNB1;GLI3;PTH;SP1;RDH10;VDR;TNFRSF21;PTN;OSR2;LRP5;TGFB3;COL1A1;COL6A1;ASPN;ZFAND5;NDST1;SMAD3;COL12A1;GDF11;PRKCA;SOX5;COL11A1;ZEB1;RECK;IGFBP4;IL6R;NPR3;COL5A2;FOXD4L1;ACVR2A;COL3A1;COL15A1;THBS2;FBN2;BMP4;ROR2;CTGF;TGFBR2;THBS1;GDF10;WNT5A;THRA;PBX1;NPR2;COL9A1;TULP3;PKDCC
neuron projection development	2.80E-11	99	745	RPS6KA3;PLCG1;LIMK1;NAV1;ATP7A;PARD3;HOXA3;COL5A1;LAMA3;LIFR;AFG3L2;DPYSL2;CDH2;LPPR4;CNTNAP1;PRTG;SRGAP1;DOCK1;CELSR3;MYO6;MAP2;SLIT2;ABLIM3;SNAP25;EPHB3;TIAM1;EXT1;AGRN;ROCK2;GLI3;PTPRF;NTN4;FYN;SEMA5A;SEMA3B;LAMC1;LAMB1;CREB1;MAP2K2;COL4A5;ENAH;ITGA5;RHOB;NEO1;COL1A1;EP300;MAP1B;COL4A1;PLXNA1;COL6A1;SOS2;AP2B1;PRKCA;CDC42;TNIK;IGF1R;RUFY3;EFNA5;NRXN3;COL6A3;MYL9;ERBB2;PLXND1;RPS6KA2;SRC;MYH9;CSNK2A2;PTPRG;CD72;COL5A2;FZD2;ROCK1;NUMBL;FOXD4L1;MAP1A;EVL;ROBO1;DISC1;CAPRIN1;EGFR;COL3A1;ABI2;SPTAN1;PIP5K1C;VCAN;PTPRA;CDH4;SRGAP2;SPOCK1;LSAMP;CNTN3;WNT5A;NCDN;NEFH;ULK1;KLF7;MYH10;COL9A1;TNC
vasculature development	4.64E-10	91	519	PPAP2B;PLCG1;ITGB8;ZFPM2;CD40;PKD1;LAMA4;ANGPTL2;VEGFB;ATP7A;TCF7L2;HOXA3;COL5A1;CD248;CDH2;FN1;ELN;ADAMTS15;HDAC5;COL18A1;NAA15;SLIT2;WARS2;GPI;DICER1;MMP14;TIPARP;TGFB2;CTNNB1;GLI3;NTN4;ZMIZ1;WARS;SEMA5A;PLCD3;EDNRA;SP1;STAT3;ACVRL1;CREB1;PTN;FZD4;WASF2;PRKD2;LRP5;ITGA5;IL22RA1;RHOB;NF1;EFNB2;COL1A1;DCBLD1;COL4A1;ECM1;ZFAND5;HRG;GPR124;CDC42;SDC3;RAD50;RECK;ERBB2;PLXND1;SRC;MYH9;FZD2;TBK1;ERAP1;ROBO1;COL3A1;COL15A1;THBS2;CYR61;COL8A2;BMP4;EPAS1;EPHB4;QKI;CTGF;MMP19;TGFBR2;THBS1;MYLK;GATA6;WNT5A;PLXDC2;MIB1;HDAC7;KRIT1;FIGF;TNC
extracellular matrix organization	4.87E-09	35	151	BCL3;LUM;FOXF2;ATP7A;PODN;COL5A1;ADAMTS2;ITGA11;COL18A1;CSGALNACT1;TGFB2;LAMC1;PXDN;DPP4;COL4A5;P4HA1;NF1;COL1A1;COL6A1;ASPN;COL12A1;COL11A1;LTBP2;RECK;COL5A2;JUND;FBLN5;COL3A1;COL5A3;CYR61;COL8A2;CTGF;CCDC80;TNC;TRAM2
muscle structure development	1.25E-05	54	392	MKL1;PPP3CA;TCF7L2;TSHZ3;AFG3L2;CDH2;HDAC5;ITGA11;SIX1;DICER1;SIN3B;SOX9;AGRN;PDZRN3;CTNNB1;ZFHX3;IGFBP5;SGCD;NEO1;EP300;CSDA;LMNA;SORT1;SMAD3;LAMA2;SEPN1;CDC42;MEF2C;ZEB1;RECK;COL6A3;ERBB2;MYH9;MBNL2;TPM1;EHBP1;POU6F1;SVIL;TSC1;COL5A3;TGFBR3;BMP4;TBX5;ADAM12;EPAS1;EMD;QKI;MYLK;BTBD9;GATA6;DISP1;THRA;FLNB;TNC
Wnt receptor signaling pathway	1.77E-05	48	250	PPAP2B;SNAI2;LRP1;HBP1;CALCOCO1;TCF7L2;LRP6;KREMEN1;MACF1;TBL1X;CTNNB1;GLI3;MITF;TCF4;GLIS2;FZD4;TCF7L1;TCF7;CTNNBIP1;LRP5;COL1A1;DVL3;SMAD3;DAB2;TNIK;DAB2IP;CTNND1;LATS2;TLE4;CSNK2A2;SOX4;FZD2;APCDD1;DISC1;GRB10;MCC;CHD8;TBL1XR1;EMD;ROR2;DAAM1;MESDC2;TTC12;NOTCH3;BTRC;WNT5A;LDB1;PBX1
positive regulation of cell death	3.56E-05	92	860	JMY;SERINC3;BCL3;CLIP3;C1RL;CD40;PPARG;BBC3;ARHGEF17;ERBB4;HDAC5;COL18A1;CDKN1B;SLIT2;ARHGEF3;PLK2;EPHB6;TRAF4;APPL1;TIAM1;TGFB2;CTNNB1;PPIC;PTH;ZMAT3;PTPRF;XPC;MAP3K1;VDR;FOXO3;TNFRSF21;BCL6;EP400;SET;CDK6;UNC13B;PVR;CASP10;TGFB3;TRIB2;NF1;IFI16;TNFSF4;ATF5;EP300;ACVR1B;APH1A;CADM1;SOS2;SORT1;SMAD3;NUPR1;ATXN7;PDE5A;ADAMTSL4;PRKCA;DAB2;DAB2IP;LATS2;RASSF4;PHLDA3;ERBB2;PPP3R1;SOX4;STAT5B;AKAP13;SGPL1;RBM5;THBS2;RIN2;TP53INP1;DYNLL2;LYST;FGD4;PHF17;BMP4;RNF144B;TBX5;CASP7;BRD2;RASSF2;MAP3K5;RB1CC1;CTGF;BNIP3L;THBS1;GATA6;WNT5A;HDAC7;DYRK2;DLC1;OLR1
