# Supplementary material for: Gene expression profiling following NRF2 and KEAP1 siRNA knockdown in human lung fibroblasts identifies CCL11/Eotaxin-1 as a novel NRF2 regulated gene
Source: Respir Res. 2012 Oct 12;13(1):92. doi: 10.1186/1465-9921-13-92 (PMC3546844; doi:10.1186/1465-9921-13-92)
Supplement: Additional file 3 — Down-regulated genes by NRF2 and KEAP1 siRNA knockdown. List of genes whose expression is decreased with NRF2 and KEAP1 siRNA knockdown. Genes are group based on annotated biological processes. [file 1465-9921-13-92-S3.pdf]

Similar Set	Expectation	Overlap	Set	Input Identifiers
mitotic cell cycle	6.09E-35	174	812	TSGA14;DSCC1;E2F8;BRCA1;INCENP;PLK4;NUP107;POLE2;RCC2;BCAT1;CDT1;SKP2;HMGA2;AURKB;RANGAP1;THAP1;MCM6;FGFR1OP;SKA3;HAUS2;FBXO5;RHOJ;TTF2;NUP43;HAUS1;KLHL15;TYMS;ORC1;CKAP2;CDK13;CEP78;LZTS2;MAD1L1;CDKN2D;CDCA3;TOE1;G0S2;ORC6;KIF22;CENPN;MCM2;MCM4;DDX11;CDC6;POLA2;RFC4;DBF4;CDCA5;RB1;RCL1;TAOK1;KIF20B;CCDC94;E2F1;TBRG4;CCNA1;PPP1CC;DYNC1H1;CENPL;ZWILCH;CENPH;PCNA;BCL2;HSP90AA1;ANAPC1;SEH1L;NEDD1;CEP72;GINS2;CDK11B;ITGB3BP;GSG2;UPF1;SKP1;TACC1;SFI1;NEDD9;LRRCC1;PRC1;BRCA2;TIPIN;CDKN3;SGOL1;CDC45;SMC1A;KLHL21;AURKA;MCM8;ID1;SPC24;RFC5;CDK2;MPHOSPH9;KATNB1;RASSF1;USP16;BUB1B;CEP110;PRIM1;RFWD3;RRS1;GTSE1;MAPRE1;NOLC1;DTL;RRM2;GINS1;ZNF330;KIFC1;YEATS4;FOXC1;NUP160;APC;CLSPN;SIRT7;NBN;EZH2;RFC3;CDC27;HAUS6;SMC3;AKT1;MCM5;AHCTF1;NCAPG;CEP192;PSMF1;BUB3;GINS4;CEP55;CDK1;CDC20;NSL1;BLM;MCM3;PES1;FEN1;LZTS1;UBE2I;RFC1;CEP57;CDK5RAP2;NCAPG2;CHAF1B;E4F1;PBK;PINX1;STAG1;SKA1;ANLN;ACTR1A;CENPO;SMC2;TUBGCP2;RFC2;POLD3;PKIA;MAD2L2;ODF2;MLF1IP;MUS81;KATNA1;ORC5;CCNB1;CENPA;CDC5L;CENPI;RINT1;CAV2;TUBGCP3;ZWINT;SAC3D1;XRCC2;MBD1
DNA replication	5.26E-17	77	318	DSCC1;RRM1;POLE2;CDT1;TK1;NFIB;TNKS2;MCM6;REPIN1;MED1;TYMS;ORC1;SUPT16H;TBRG1;NOL8;RALA;ORC6;TNFAIP1;MCM2;ACD;MCM4;CDC6;CDC111;POLA2;RFC4;DBF4;DEK;PCNA;EME1;GINS2;PPIA;UPF1;BRCA2;CHEK1;TIPIN;CDC45;RECQL;SMC1A;MCM8;MMS22L;DCLRE1B;RFC5;CDK2;UNG;XRCC4;PRIM1;DNAJA3;DTL;RRM2;GINS1;ING5;TMPO;CLSPN;SLBP;RECQL5;NBN;RFC3;TOP3A;SMC3;MCM5;GINS4;CDK1;BLM;MCM3;FEN1;RFC1;CHAF1B;E4F1;RFC2;POLD3;MSH3;MUS81;ORC5;SHC1;DUT;TERF2;RBM14
regulation of cell cycle arrest	1.78E-15	59	275	BRCA1;INCENP;RCC2;CDT1;MCM6;FANCG;ORC1;MAD1L1;ORC6;MCM2;MCM4;CDC6;RFC4;DBF4;RB1;TAOK1;ZWILCH;PCNA;ANAPC1;BRCA2;CHEK1;TIPIN;RBBP8;CDC45;SMC1A;MCM8;BRCC3;DCLRE1B;RFC5;CDK2;RASSF1;BUB1B;RFWD3;GTSE1;DTL;APC;CLSPN;NBN;RFC3;CDC27;MCM5;PSMF1;BUB3;CDK1;CDC20;BLM;MCM3;PES1;CDK5RAP2;GPS2;H2AFX;RFC2;MAD2L2;MSH3;MUS81;ORC5;CCNB1;RINT1;ZWINT
regulation of cell cycle process	1.50E-12	85	439	BRCA1;INCENP;PLK4;RCC2;CDT1;MCM6;FANCG;FBXO5;ORC1;CKAP2;CDK13;MAD1L1;CTCF;ORC6;MCM2;MCM4;CDC6;RFC4;DBF4;CDCA5;RB1;TAOK1;KIF20B;ZWILCH;PCNA;ANAPC1;NEDD1;EME1;GSG2;UPF1;PRC1;BRCA2;CHEK1;TIPIN;RBBP8;CDC45;SMC1A;MCM8;BRCC3;DCLRE1B;RFC5;CDK2;MPHOSPH9;RASSF1;BUB1B;RFWD3;GTSE1;DTL;APC;CLSPN;SLBP;NBN;RFC3;SFRP1;CDC27;SMC3;MCM5;CEP192;PSMF1;BUB3;CDK1;CDC20;BLM;MCM3;PES1;FEN1;LZTS1;CDK5RAP2;CHAF1B;E4F1;GPS2;ANLN;H2AFX;RFC2;PHC3;PKIA;MAD2L2;MSH3;MUS81;ORC5;CCNB1;CDC5L;RINT1;CAV2;ZWINT
DNA repair	1.74E-10	72	368	BRCA1;POLE2;ZNF180;FANCG;USP1;TYMS;SUPT16H;MUM1;CDKN2D;UBE2T;KIF22;C9orf80;RFC4;CDCA5;APEX2;RAD51C;PCNA;EME1;GADD45G;UPF1;BRCA2;CHEK1;RAD54L;RBBP8;RECQL;SMC1A;TSN;MCM8;BRCC3;MMS22L;DCLRE1B;RFC5;UNG;XRCC4;POLR2E;RFWD3;DTL;FANCE;CLSPN;RECQL5;NBN;RFC3;SMC3;RAD18;MND1;BLM;FEN1;FANCB;BCCIP;RFC1;CHAF1B;GTF2H3;H2AFX;PRKDC;CEBPG;RAD54B;RFC2;POLD3;MAD2L2;PARP1;TOPBP1;SMC5;MSH3;MUS81;TDP1;NONO;OGG1;RUVBL2;RBM14;XRCC2;MBD1;USP10
cytoskeleton organization	2.48E-10	107	881	ARHGAP11B;BRCA1;INCENP;PLK4;RCC2;RANGAP1;TRIM36;FGFR1OP;SKA3;HAUS2;FBXO5;RHOJ;HAUS1;CKAP2;CORO1A;EPB41L2;SGCB;MAP4;RALA;VIL1;TNFAIP1;IQGAP1;PACSIN2;ARHGAP22;PDXP;DYNC1H1;CENPH;BCL2;SEH1L;NEDD1;CEP72;CAPG;ARHGAP17;FKBP4;BICD2;SKP1;PDLIM7;KIRREL;TACC1;SFI1;PDLIM5;NEDD9;PLS1;PRC1;BRCA2;SGOL1;SMC1A;RHOB;TRPM7;ADD1;AURKA;GEM;ABLIM1;KATNB1;CALD1;RASSF1;BUB1B;FSD1L;MAPRE1;EPB41L3;INF2;APC;KBTBD2;CLIC4;EZH2;TAGLN3;SFRP1;HAUS6;SMC3;BAX;CAP1;CEP192;MAST1;USP6;EZR;BUB3;CEP55;SRF;AFAP1;LIMA1;LZTS1;NES;CDK5RAP2;RAP1B;MYH11;SKA1;WASH1;ANLN;SDC4;TUBGCP2;ARL8B;SPRED2;TRIP6;ODF2;TRIOBP;ACTA1;KATNA1;RASA1;CCNB1;CENPA;ARPC5L;RINT1;TUBGCP3;ZWINT;PDPK1;SAC3D1;SUN1
cell cycle checkpoint	5.56E-10	58	267	BRCA1;INCENP;RCC2;CDT1;MCM6;FANCG;ORC1;MAD1L1;ORC6;MCM2;MCM4;CDC6;RFC4;DBF4;RB1;TAOK1;ZWILCH;PCNA;ANAPC1;BRCA2;CHEK1;TIPIN;RBBP8;CDC45;SMC1A;MCM8;BRCC3;DCLRE1B;RFC5;CDK2;BUB1B;RFWD3;GTSE1;DTL;APC;CLSPN;NBN;RFC3;CDC27;MCM5;PSMF1;BUB3;CDK1;CDC20;BLM;MCM3;PES1;CDK5RAP2;GPS2;H2AFX;RFC2;MAD2L2;MSH3;MUS81;ORC5;CCNB1;RINT1;ZWINT
response to DNA damage stimulus	2.34E-09	97	603	BRCA1;POLE2;CDT1;HMGA2;ZNF180;FANCG;USP1;MAPK1;TYMS;SUPT16H;MUM1;CDKN2D;UBE2T;KIF22;ACD;C9orf80;RFC4;CDCA5;APEX2;RAD51C;EEF1E1;DEK;PCNA;BCL2;EME1;GADD45G;UPF1;BRCA2;CHEK1;RAD54L;TIPIN;RBBP8;ALKBH8;RECQL;SMC1A;TSN;MCM8;BRCC3;MMS22L;DCLRE1B;RFC5;CDK2;UNG;RASSF1;FBXO45;XRCC4;POLR2E;RFWD3;GTSE1;DTL;ING5;APC;FANCE;CLSPN;RECQL5;NBN;RFC3;SMC3;BAX;RAD18;PSMF1;MND1;CDK1;MAPK3;BLM;FEN1;FANCB;BCCIP;RFC1;CHAF1B;GTF2H3;GPS2;H2AFX;PRKDC;CEBPG;RAD54B;RFC2;POLD3;MAD2L2;PARP1;PPP1R15A;TOPBP1;SMC5;MSH3;MUS81;TDP1;NONO;ZNF793;TERF2;RINT1;OGG1;RUVBL2;MAPK14;RBM14;XRCC2;MBD1;USP10
DNA recombination	7.97E-08	44	192	BRCA1;POLE2;POLA2;RFC4;APEX2;RAD51C;PCNA;EME1;PPIA;BRCA2;CHEK1;RAD54L;RECQL;TSN;RBPJ;MMS22L;DCLRE1B;RFC5;UNG;XRCC4;PRIM1;RECQL5;NBN;RFC3;TOP3A;RAD18;MND1;BLM;FEN1;RFC1;PSMC3IP;H2AFX;PRKDC;RAD54B;HMGB3;RFC2;POLD3;SMC5;MSH3;MUS81;NONO;RUVBL2;RBM14;XRCC2
