# Supplementary figures and images for: Gene expression profiling following NRF2 and KEAP1 siRNA knockdown in human lung fibroblasts identifies CCL11/Eotaxin-1 as a novel NRF2 regulated gene
Source: Respir Res. 2012 Oct 12;13(1):92. doi: 10.1186/1465-9921-13-92 (PMC3546844; doi:10.1186/1465-9921-13-92)

Additional file 4

A

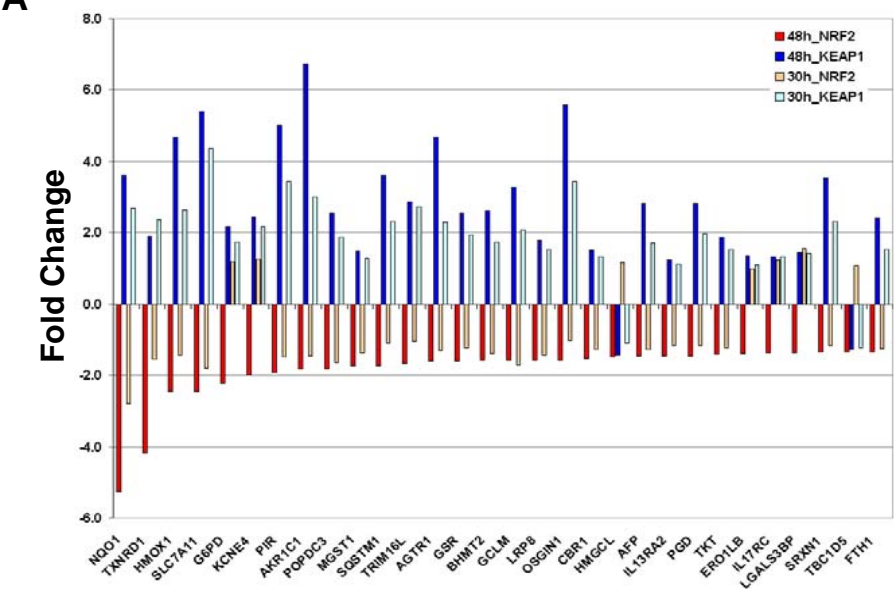

B

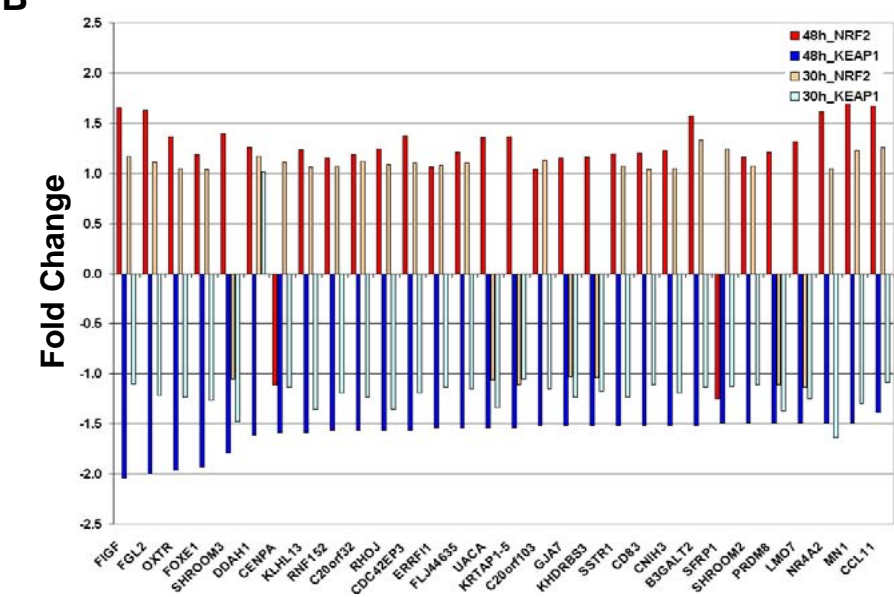

Supplement: Additional file 4 — Most significant NRF2- and KEAP1-modulated anti-correlated genes identified in microarray studies. Figure displaying (A) Top 30 genes knockdown by NRF2 siRNAs at 48 hours. Genes were sorted based on their fold change knocked down by NRF2 siRNAs at 48 hours. The corresponding fold changes modulated by NRF2 siRNAs at 30 hours and KEAP1 siRNAs at 48 and 30 hours are also shown. (B) Genes significantly knock down by KEAP1 siRNAs at 48 hours. Genes were sorted based on their fold change knocked down by KEAP1 siRNAs at 48 hours. The corresponding fold changes modulated by NRF2 siRNAs at 30 and 48 hours and KEAP1 siRNAs at 30 hours are also shown. CCL11/eotaxin is included in the Figure as a reference although it is not one of the top 30 KEAP1 siRNAs knock down genes. [file 1465-9921-13-92-S4.pdf]

Additional file 6

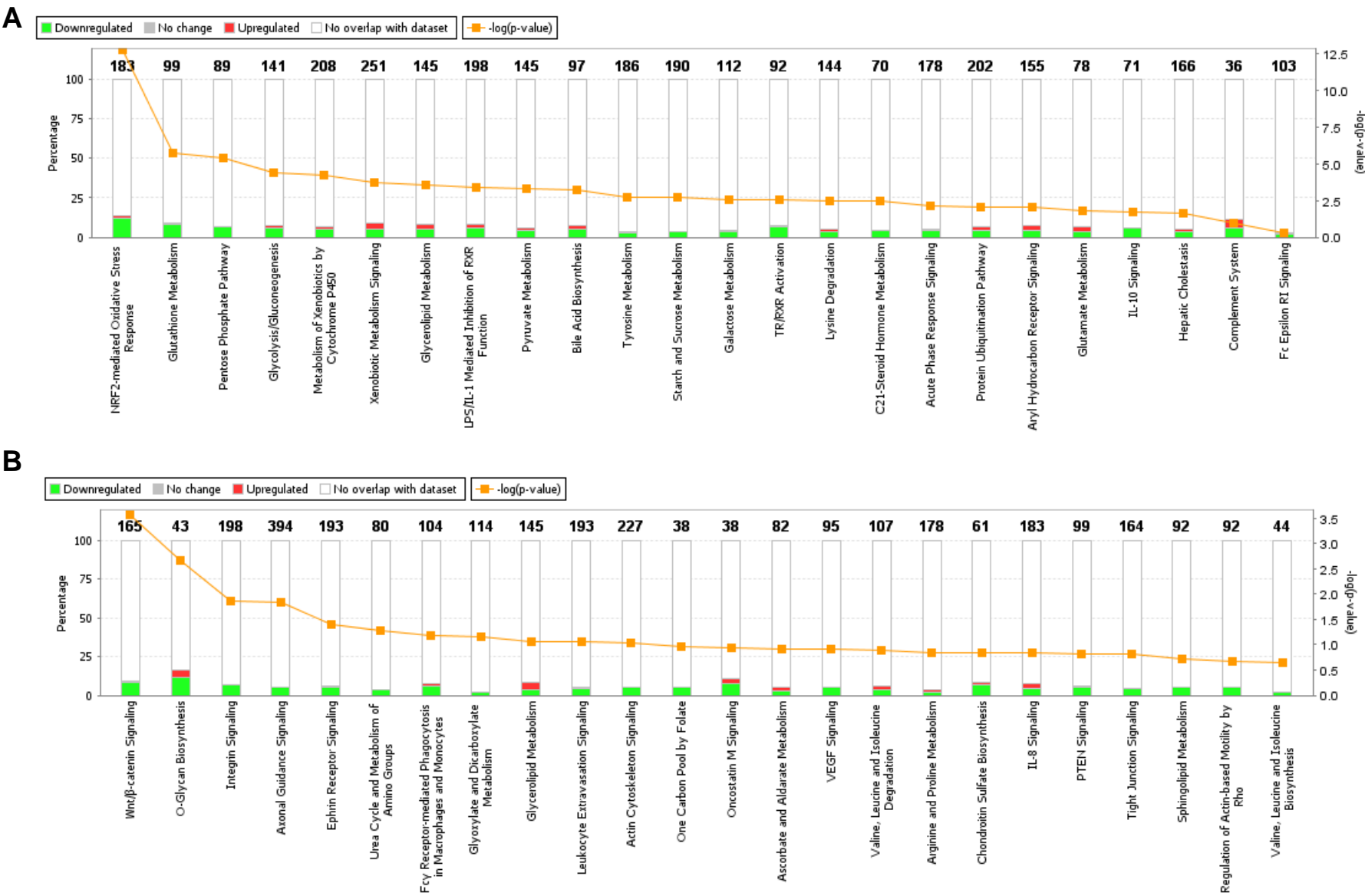

Supplement: Additional file 6 — Ingenuity pathway analysis of anti-correlated genes knock-down by (A) NRF2 siRNAs or (B) KEAP1 siRNAs. Ingenuity pathway analysis of anti-correlated genes knock-down by (A) NRF2 siRNAs or (B) KEAP1 siRNAs. Of the 1,045 anti-correlated signature genes, 361 sequences down-regulated by NRF2 siRNAs and 684 sequences down-regulated by KEAP1 siRNAs were individually up-loaded onto Ingenuity Pathway Analysis (IPA) tool for querying canonical pathways associated with the input gene sets (http://www.ingenuity.com). [file 1465-9921-13-92-S6.pdf]
