# Supplementary material for: Gene expression profiling following NRF2 and KEAP1 siRNA knockdown in human lung fibroblasts identifies CCL11/Eotaxin-1 as a novel NRF2 regulated gene
Source: Respir Res. 2012 Oct 12;13(1):92. doi: 10.1186/1465-9921-13-92 (PMC3546844; doi:10.1186/1465-9921-13-92)
Supplement: Additional file 5 — Annotation of anti-correlated NRF2 and KEAP1 siRNA knock-down gene set at 48 hours. List of genes whose expression is modulated in an anti-correlated direction with NRF2 and KEAP1 siRNA knockdown. Genes are group based on annotated biological processes. [file 1465-9921-13-92-S5.pdf]

Annotation of anti-correlated *NRF2* and *KEAP1* siRNA knock-down gene set at 48 hours.

Similar Set	Expectation	Source	Overlap	Set	Input Identifiers
actin cytoskeleton organization and biogenesis	4.35E-05	GO Biological Process	52	513	FARP1;DLC1;TRIOBP;CNN1;CNN2;MAPK14;CTGF;AMOT;CD55;EPB41L2;F2R;F2RL1;FAT;FHL1;FLNB;FLT1;MACF1;GNA12;FHOD1;FMN1;SHROOM2;ILK;ARF6;LASP1;LGALS8;LMO7;PPP1R12A;NEDD9;PAK2;LIMA1;ENAH;FMN2;PTGER4;RHOJ;PTPRK;RAC1;CCL11;SDC4;SPTBN1;VASP;VCL;ZYX;FHOD3;MED28;DIAPH3;CAST;NCK2;PKP4;PSTPIP2;PSCD2;PDE4DIP;GIT2
NRF2-mediated Oxidative Stress Response	0.000194	Ingenuity Pathways	23	168	MAPK14;NQO1;EPHX1;FTH1;FTL;GCLM;GSR;GSTM3;GSTM4;GSTM5;UBE2K;HMOX1;MGST1;PRDX1;SOD1;ACTG2;TXN;TXNRD1;CAT;CBR1;SQSTM1;USP14;RBX1
blood vessel development	0.00036	GO Biological Process	46	456	GJC1;EDIL3;CITED2;SPON1;HTATIP2;ANTXR2;CTGF;AMOT;AFP;EFNB2;EGR1;EPHA2;EPAS1;F2R;F2RL1;FGF7;FIGF;FOXO1;FLT1;DDAH1;GJA1;GPC1;HMOX1;NR4A1;IFI16;KCNMA1;MEF2C;MIF;EGFL7;PLAU;PTGIS;WFDC1;RAC1;CCL11;TNS3;BMP4;TEK;NR2F2;TIMP3;TXN;VASP;MED28;APOLD1;CAV1;ANGPTL1;HS6ST1
oxidation reduction	0.000403	GO Biological Process	52	549	HTATIP2;MTHFD2;ADH1C;ADH5;SRXN1;AKR1C1;AKR1C2;NQO1;ALDH3A1;ALDH1B1;ALDH1A3;LTB4DH;FBXL11;AKR1B1;FTH1;KDSR;MSRB3;G6PD;MTHFD1L;GSR;CYP2S1;HCCS;HMOX1;HSD11B1;FADS1;FADS3;ME1;ASPH;PRDX1;CYB5R2;PGD;PLOD2;GFOD1;POR;EGLN1;ERO1LB;AKR1B10;ALDH18A1;SQRDL;QDPR;NXN;BLVRA;SOD1;TXNRD1;UGDH;ACOX3;CAT;AIFM2;ADO;CBR1;CBR3;FADS2
Heme metabolism	0.00152	GeneGo Pathways	9	28	ALAS1;FECH;FTH1;FTL;HCCS;HMOX1;QDPR;BLVRA;CAT
regulation of cell motility	0.0256	GO Biological Process	39	420	DLC1;ABHD2;CNN1;CNN2;MAPK14;CTGF;AMOT;AGTR1;EFNB2;EPAS1;F2R;F2RL1;FGF7;DDAH1;CLIC4;DKK3;FHOD1;LGALS8;PPP1R12A;NEDD9;OXTR;EGFL7;TTRAP;PTGER2;PTGER3;PTPRK;PVR;WFDC1;RAC1;SORT1;SDC4;SKI;SORL1;SSTR1;NR2F2;VASP;VCL;NEXN;GIT2
Metabolism of Xenobiotics by Cytochrome P450	0.115	Ingenuity Pathways	13	93	ADH1C;ADH5;AKR1C1;AKR1C2;EPHX1;ALDH3A1;ALDH1A3;GSTM3;GSTM4;GSTM5;CYP2S1;MGST1;CSGALNACT1
O-Glycan Biosynthesis	0.173	Ingenuity Pathways	7	28	GALNT5;GALNTL2;FUT4;GALNT2;GALNT7;ST3GAL4;GALNT4
induction of apoptosis	0.217	GO Biological Process	54	720	DLC1;CEBPB;HTATIP2;MAPK14;CTGF;DAP;AFP;DUSP6;EPHA2;F2R;FHL2;PHLDA1;DKK1;RIPK5;GAS1;DKK3;BIN1;HIPK2;HMOX1;NR4A1;IFI16;FAS;ARF6;LTBR;NEDD9;PAK2;POLB;EIF2AK2;PTGIS;PVR;RAC1;SORT1;SDC2;BMP4;SNAI1;SOD1;SOX4;BOK;SRF;BRCA2;STAT1;TALDO1;TIMP3;TXNRD1;XPC;PLEKHF1;ULBP1;CABLES2;CUL1;AIFM2;RUNX3;GPRC5A;RASSF2;BCLAF1
enzyme linked receptor protein signaling pathway	0.296	GO Biological Process	54	730	SPRY1;CITED2;TACC2;PDLIM5;CNN1;CSF1;CTGF;BMPER;AGTR1;EPHA2;F2R;F2RL1;FGF7;FIGF;FOXO1;FLT1;SNF1LK2;MSTN;GPC1;RGMB;HIPK2;IL1RAP;ILK;KIT;DDR2;DOK5;ERBB2IP;PTGER2;PTGER4;PTPRK;RAC1;BDNF;SORT1;CCL11;SDC2;GREM2;SMURF2;TNS3;SKI;BMP4;TEK;TIMP3;CLEC3B;NR4A3;SHOC2;NCK2;CAV1;PDLIM4;RUNX2;RUNX3;PCAF;SQSTM1;ANGPTL1;CEP57
skeletal development	0.312	GO Biological Process	32	359	CDH11;COL10A1;COL13A1;CSF1;CTGF;BMPER;ETS2;GPC4;DKK1;FOXE1;FLNB;GPC1;MEF2C;PRDX1;PRELP;SRGN;FEM1C;PTGER4;PTGIS;RYK;SORT1;SKI;BMP4;SNAI1;SOX9;STAT1;TCF3;TLE1;CLEC3B;TWIST1;PDLIM4;RUNX2
response to oxidative stress	0.361	GO Biological Process	29	315	PSIP1;DDIT4L;SRXN1;AKR1C1;NQO1;CLN8;FH;FBXW11;SLC7A11;FTH1;FTL;GCLM;GSR;GSTM3;HMOX1;IFI16;MGST1;PRDX1;GLRX5;PLOD2;EGLN1;QDPR;RAC1;SOD1;TXN;TXNRD1;CAT;PNPT1;SQSTM1
Pentose phosphate pathway/ Rodent version	0.41	GeneGo Pathways	5	15	G6PD;GPI;PGD;TALDO1;TKT
steroid metabolic process	0.439	GO Biological Process	28	304	NR1H3;OSBPL8;ADH1C;CRH;SNF1LK;AKR1C1;AKR1C2;AFP;CLN8;OSBPL3;HSD11B1;KCNMA1;STS;CYB5R2;ATP8B1;POR;AKR1B10;PTGER2;SET;SOD1;SORL1;STAR;NR2F1;NR2F2;UGDH;ACOX3;EBPL;CAV1
Glycerolipid Metabolism	0.507	Ingenuity Pathways	11	82	MGLL;ADH1C;ADH5;ALDH3A1;ALDH1B1;ALDH1A3;AKR1B1;FUT4;GK;GLA;AKR1B10

Gene set annotation analysis was performed by querying GO biological process, GeneGo and Ingenuity pathways using 893 anti-correlated Nrf2 and Keap1 siRNA knock-down genes obtained at 48 hour time point.
